# Compulsory Psychiatric Admission in a Patient With Metastatic Breast Cancer: From Palliative Care to Assisted Suicide

**DOI:** 10.3389/fpsyt.2020.00454

**Published:** 2020-05-25

**Authors:** Felix Noppes, Egemen Savaskan, Florian Riese

**Affiliations:** ^1^Department of Geriatric Psychiatry, Psychiatric University Hospital Zurich, Zurich, Switzerland; ^2^University Research Priority Program “Dynamics of Healthy Aging”, University of Zurich, Zurich, Switzerland

**Keywords:** palliative care, assisted suicide, coercion, involuntary admission, compulsory commitment, psycho-oncology, concealed medication, covert medication

## Abstract

The provision of palliative care in psychiatry and the use of coercion in palliative care are underexplored areas. We report the case of a 65-year-old woman with cerebral metastatic breast cancer who was compulsorily admitted from a specialized palliative care ward to a psychiatric inpatient ward in Zurich, Switzerland. While in specialized inpatient palliative care, the patient had resisted palliative care but was found to lack decision-making capacity for her treatment due to disordered thought process and paranoid delusions. Under our care, which involved coercive treatment in the form of concealed administration of an antipsychotic, the patient's psychiatric symptoms improved. She regained decision-making capacity, was granted discharge from hospital, and ended her life by assisted suicide on the day of discharge.

## Case Description

A 65-year-old widow was diagnosed with cerebral metastatic breast cancer after experiencing a generalized seizure. Following this initial diagnosis, the patient repeatedly discussed the different care options and their possible impact on survival time, quality of life, symptom load, and personhood with her son. Through these discussions, it became clear that she valued “remaining herself” highest, i.e. being awake and conscious and living and dying as autonomously as possible, and the patient decided against further diagnostic workup and aggressive treatment. She did not make a written advanced directive. She was admitted to a general hospital palliative care ward in Zurich, Switzerland. Among other palliative measures, corticosteroid treatment to lower intracranial pressure was started and the patient was discharged home with treatment by a palliative homecare team. However, the patient developed paranoid delusions and disordered thought process, and thus stopped taking corticosteroids.

The patient was member of a Swiss assisted suicide organization—highlighting her general tendency towards valuing autonomy at the end of life. From home, the patient contacted the organization with the wish to commit assisted suicide but eventually reconsidered since she was unable to set an exact date for the procedure and thus felt that she was “not ready yet”. Consequently, the patient did not commit assisted suicide at this point. Due to the psychiatric symptoms, adequate palliative care (including local wound care for the exulcerated primary tumor in her right breast) could not be provided at home and the patient was readmitted to the general hospital palliative care ward. However, due to her psychiatric symptoms, the patient was also unable to cooperate in nursing and medical care in the palliative inpatient setting. Six weeks after the initial seizure, she was compulsorily admitted to our old age psychiatry ward with the aim of meeting her psychiatric and palliative care needs.

Upon admission to our ward, the patient presented with an exulcerated tumor in the right breast, a dilated right pupil, and a generalized itch. The patient refused to undergo a physical exam (or even be touched) upon admission and throughout the hospitalization, so assessment of physical symptoms was based on observation from a distance only. The patient reported no pain. She had paranoid delusions and a disordered thought process and appeared anxious. She had no insight into her physical and psychiatric condition and her prognosis. As a consequence of her delusions, she continued to perform self-harmful manipulations on the primary tumor. The patient declined all medication and did not allow nurses to perform basic care including wound care for the tumor. She generally stayed in her single room, refused to eat, and she only drank minor quantities from previously unopened softdrink bottles. According to the previous medical documentation and the patient's son, she had no prior history of mental illness. The patient did not fulfill diagnostic criteria for delirium. A diagnosis of organic delusional disorder was made (ICD-10: F06.2, corresponding to DSM-5 “psychotic disorder due to another medical condition”). Due to her disordered thought process and delusions, the patient was found to lack decision-making capacity for her medical care.

According to the patient's son, who had surrogate healthcare decision-making power according to Swiss law, the patient had been aware of her terminal condition before and had repeatedly stated that she was strictly against any form of sedation. A joint decision was made with the patient's son for concealed administration of an antipsychotic with low sedating properties (risperidone) in the patient's preferred soft drink. We attempted to administer a daily dose of 2 mg risperidone per day. However, since the patient did not consume reliable quantities of her softdrink, we estimated the ingested dose to be lower than 1 mg per day. No plasma levels could be measured, since the patient refused blood sampling and we decided that coercive blood sampling was not indicated. Despite the patient's obvious anxiety, we decided to withhold benzodiazepines unless further seizures would occur since we could not guarantee non-sedating dosing due to the concealed administration.

Either due to spontaneous fluctuations in symptoms, decreasing detrimental effect of steroids on mental state after their discontinuation, antipsychotic treatment or a combination of all of these, the patient's thought disorder improved drastically three weeks into the treatment and she became able to coherently and repeatedly express her wish to die by assisted suicide. According to the patient, this wish was mainly explained by her intent to conclude her life autonomously and consciously. With the help of her son, the patient initiated the procedure for assisted suicide. A comprehensive capacity evaluation was performed by the same psychiatrist who had also assessed the patient upon her initial request for assisted suicide and had determined her to lack decision-making capacity. The psychiatrist was independent from our institution and experienced in capacity evaluations. At this point, the patient was found to have regained decision-making capacity. Antipsychotic treatment was thus discontinued and the patient was granted discharge from the hospital as she continued to refuse psychiatric care. The patient committed assisted suicide at home by ingesting a lethal dose of a barbiturate on the same day of discharge.

## Discussion

This case of a patient who resisted palliative care but lacked decision-making capacity and eventually ended her life by assisted suicide highlights several controversial issues at the interface of palliative care and psychiatry. First, is psychiatry the right place to perform palliative care in such cases? Second, is palliative care at all compatible with coercive care? And third, how does assisted suicide relate to psychiatry?

To the first question, if psychiatry is the right place to perform palliative care for patients that have both palliative care needs and psychiatric symptoms that interfere with care: palliative care and psychiatry often work well together—most notably in the field of psycho-oncology (1–3). However, while psychiatrists are experts in mental health and in treating patients with impaired decision-making capacity, only few psychiatrists are trained or even certified in palliative care (4). Furthermore, psychiatric services may be located outside general hospitals that could perform certain palliative emergency procedures. Finally, the adequate reimbursement of palliative care in psychiatric institutions is an unresolved issue. For example, psychiatric services may be precluded from using billing codes for palliative care depending on local regulations. On the other hand, specialized psychiatric services are obviously competent in the treatment of psychiatric disorders: In palliative care cases where psychiatric symptoms are leading, patient needs may be best addressed there. Furthermore, psychiatric services are experts in treating disruptive behaviour that may interfere with palliative care in other settings.

In this case, neither discharge home nor hospice placement was deemed possible and the patient behaved too disruptively to continue treatment in a palliative care unit. The referring palliative care specialists thus saw psychiatry's experience with involuntary treatments as the only remaining option to reduce patient suffering. Eventually, provision of high-quality palliative care is possible where the required expertise is available. In order to be an option for patients with altered mental state and palliative care needs, inpatient psychiatric services will therefore have to invest in capacity building in this area.

Second, is palliative care at all compatible with coercive care? Supporting patient autonomy is often considered a mainstay of palliative care—even though it may be difficult to achieve (5). On the other hand, the Swiss criteria for a compulsory psychiatric commitment were formally fulfilled (i.e. presence of a psychiatric disorder, risk of acute self-harm, placement to a suitable treatment institution). We tried to follow patient preferences by withholding sedation, foregoing nursing care against resistance, and later by discharging the patient. Still, coercion was used in the form of concealed medication and keeping the patient in hospital even though she generally refused care. Concealed administration of medication is associated with numerous ethical and practical issues (5, 6). In Switzerland, it is legal but considered a form of coercive treatment. As such, it is restricted to a clearly defined set of clinical situations and is subject to specific regulations (6). Among other prerequisites, adequate attempts have to be made to fully inform patients and/or their surrogate decision-makers about the planned coercive treatment both orally and in writing (as was done in this case). Despite such efforts, concealed administration remains quintessentially covert in the instant of administration, since nurses do not openly present the medication.

Treatment teams may be divided whether coercive measures are appropriate at all and especially in the suffering patient at the end of life. We tried to address these tensions by tailoring an individualized strategy in repeated discussions with the patient and her family as well as in interprofessional case conferences and team supervision with a clinical ethicist. Eventually, decision-making capacity was restored either through the natural course of disease, due to the absence of steroids or indeed due to the concealed administration of antipsychotic medication. Our coercive measures may thus have at least contributed to the patient regaining greater autonomy and enabling her to commit assisted suicide. Irrespective of psychiatric involvement, the use of coercive measures will always require individualized deliberation and careful justification balancing the harms versus benefits and the impact on patient suffering.

Third, how does assisted suicide relate to psychiatry? In Switzerland, assisted suicide is legal if assistance is offered without selfish motive to a person with decision-making capacity (7). It is further regulated by medical-ethical guidelines issued by the Swiss Academy of Medical Sciences (8). In most cases, assisted suicide is performed with the assistance of a right-to-die organization, the biggest of which had 120,000 paying members in 2018 (9) corresponding to approximately 1.5% of the Swiss population. In 2016, the Swiss Federal Statistical Office recorded 928 cases of assisted suicide representing 1.4% of all deaths in that year (10).

While assisted suicide may thus be a widespread practice in Switzerland, it remains rare in the context of mental illness. The most recent data by the Swiss Federal Statistical Office indicate that only 3% of cases had a comorbid depression, and 0.8% a dementia (11). Assisted suicide is particularly controversial in mental illness due to the concerns regarding impaired decision-making capacity. Furthermore, prevention of suicide due to mental illness is a core task of psychiatry and deeply engrained in the professional identity of psychiatrists. In line with this, about half of the psychiatrists in a recent Swiss survey did not support access to assisted suicide in cases of severe and persistent mental illness—even when decision-making capacity was maintained (12). The legal situations and specific clinical challenges for psychiatry associated with medical assistance in dying have been the topic of a recent review (13). Notably, guidelines for clinical and ethical decision-making specifically in psychiatric patients requesting euthanasia in Belgium were also recently published (14).

In this case, our main line of reasoning was that—just like in other fields of medicine—medical interventions are only justified (and legal according to Swiss law) in a competent patient when consent is provided. When the patient's thought disorder improved and she regained decision-making capacity regarding her treatment, she refused further medical care. To prolong her involuntary admission under these premises would have been unethical and—we assume—would also have been deemed illegal in court. Our responsibility was with the quality of clinical care and due process regarding capacity evaluation. The decision for assisted suicide after discharge was the competent patient's to make—not ours. However, we appreciate that maintaining this stance was probably easier in a patient with medically very limited remaining life-expectancy.

For the son, the guiding principles as surrogate decision-maker were the patient's wish of being awake and conscious, maintaining her privacy, and having an active and self-determined role in her own end of life. The major challenges in representing the patient's will arose from the multitude of individuals, professions, organizations, and institutions involved in her care. The different providers had different perspectives regarding treatment and prognosis, varied in quality of communication, reflected differently on their role in the care process, and showed different or even contradictory attitudes towards supporting patient autonomy as expressed by her wish to commit assisted suicide. For example, the patient's oncologist had issued a prescription for the lethal barbiturate on an outpatient basis. The same oncologist—then in the function as treating inpatient physician—refused to further support assisted suicide due to a hospital policy that precluded assisted suicide on hospital grounds. The patient's son considers the psychiatric inpatient treatment a success since patient preferences were observed as much as possible. However, he would have preferred to have received counselling support throughout the process so that he could have discussed the situation with somebody outside the family and not involved in the actual care process.

Concerning the strengths of this case report, we are convinced that it uniquely condenses and combines challenging areas in psychiatry: coercive treatments in palliative care situations and assisted suicide in the mentally ill. We also consider it a particular strength that the patient's son was directly involved in the writing and is acknowledged as co-author (albeit unnamed to protect patient privacy). This case report is limited by reporting a single case in a specific cultural and legal context, thus limiting the generalizability to other cases. Yet, we hope that it stimulates the clinical and ethical discussion surrounding end-of-life issues at the interface of psychiatry and palliative care. We believe that the frequency of such clinical challenges will only increase in the future.

## Ethics Statement

Written informed consent was obtained from the individual(s) for the publication of any potentially identifiable images or data included in this article.

## Author Contributions

FN and FR wrote the first draft of the manuscript. ES provided feedback and contributed to later versions of the manuscript. All authors made an important intellectual contribution to the manuscript. All authors proofread the final manuscript and consented to its submission for publication. All authors were involved in the care of the patient reported here.

## Funding

The fees for open access publication were covered by the University of Zurich.

## Conflict of Interest

The authors declare that the research was conducted in the absence of any commercial or financial relationships that could be construed as a potential conflict of interest.
